# Resting‐state neural activity and cerebral blood flow alterations in type 2 diabetes mellitus: Insights from hippocampal subfields

**DOI:** 10.1002/brb3.3600

**Published:** 2024-07-10

**Authors:** Mingrui Li, Yifan Li, Xin Tan, Chunhong Qin, Yuna Chen, Yi Liang, Shijun Qiu, Jie An

**Affiliations:** ^1^ Department of Radiology The First Affiliated Hospital of Guangzhou University of Chinese Medicine Guangzhou China; ^2^ State Key Laboratory of Traditional Chinese Medicine Syndrome Guangzhou China; ^3^ Department of Magnetic Resonance Imaging Zhanjiang First Hospital of Traditional Chinese Medicine Zhanjiang China

**Keywords:** brain function, cerebral perfusion, hippocampal subfields, resting state, typeType 2 diabetes mellitus

## Abstract

**Objective:**

In this study, multimodal magnetic resonance imaging (MRI) imaging was used to deeply analyze the changes of hippocampal subfields perfusion and function in patients with type 2 diabetes mellitus (T2DM), aiming to provide image basis for the diagnosis of hippocampal‐related nerve injury in patients with T2DM.

**Methods:**

We recruited 35 patients with T2DM and 40 healthy control subjects (HCs). They underwent resting‐state functional MRI (rs‐fMRI), arterial spin labeling (ASL) scans, and a series of cognitive tests. Then, we compared the differences of two groups in the cerebral blood flow (CBF) value, amplitude of low‐frequency fluctuation (ALFF) value, and regional homogeneity (ReHo) value of the bilateral hippocampus subfields.

**Results:**

The CBF values of cornu ammonis area 1 (CA1), dentate gyrus (DG), and subiculum in the right hippocampus of T2DM group were significantly lower than those of HCs. The ALFF values of left hippocampal CA3, subiculum, and bilateral hippocampus amygdala transition area (HATA) were higher than those of HCs in T2DM group. The ReHo values of CA3, DG, subiculum, and HATA in the left hippocampus of T2DM group were higher than those of HCs. In the T2DM group, HbAc1 and FINS were negatively correlated with imaging characteristics in some hippocampal subregions.

**Conclusion:**

This study indicates that T2DM patients had decreased perfusion in the CA1, DG, and subiculum of the right hippocampus, and the right hippocampus subiculum was associated with chronic hyperglycemia. Additionally, we observed an increase in spontaneous neural activity within the left hippocampal CA3, subiculum, and bilateral HATA regions, as well as an enhanced local neural coordination in the left hippocampal CA3, DG, HATA, and subiculum among patients with type 2 diabetes, which may reflect an adaptive compensation for cognitive decline. However, this compensation may decline with the exacerbation of metabolic disorders.

## Graphical abstract

The hippocampal subregions in patients with T2DM exhibit reduced perfusion and increased local spontaneous neural activity and coordination.

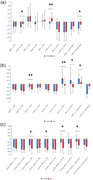

## 1 INTRODUCTION

Diabetes has become one of the most serious and common chronic diseases (Sun et al., 2022). Metabolic disorders such as hyperglycemia and insulin resistance caused by type 2 diabetes mellitus (T2DM) induce pathophysiological changes in the central nervous system, thus affecting the cognitive function of patients (Y. Li et al., 2021, 2022) and seriously affecting the quality of life of patients and causing significant social and economic burden.

The hippocampus is one of the brain regions with a high density of insulin receptors (Schulingkamp et al., 2000), and its insulin signaling pathway is closely linked to the structure and function. As a result, the hippocampus is particularly vulnerable to the effects of central insulin resistance compared to other brain regions (Biessels & Reagan, 2015; Kullmann et al., 2020; Y. Li et al., 2020). Additionally, previous research has demonstrated that the hippocampus is a particularly susceptible brain region, which is more vulnerable to damage, such as severe ischemia and hypoxia, compared to other areas in the brain (Cervós‐Navarro & Diemer, 1991; McEwen, 1997). Consequently, the hippocampus is a significant target area for secondary brain injury resulting from T2DM.

The hippocampus is a complex and heterogeneous structure that can be divided into multiple subfields, and it forms a triple synaptic loop inside (Small et al., 2011). A meta‐analysis (Biessels & Reagan, 2015) has demonstrated that T2DM can affect hippocampal structural integrity, leading to reduced cell proliferation and neurogenesis in the dentate gyrus (DG) in rat models induced by a high‐fat diet (HFD). A subsequent study has found that HFD‐induced neurogenesis reduction is associated with spatial learning and memory impairment, while reduced dendritic spine density in cornu ammonis area 1(CA1) pyramidal neurons is associated with spatial learning impairment in HFD rats. Similarly, our previous study has shown that T2DM patients exhibit decreased volume of multiple hippocampal subfields and that the abnormal structure of these subfields is associated with memory function decline (M. Li et al., 2021).

However, previous studies have paid insufficient attention to the changes in hippocampal subfields perfusion and function in T2DM patients. Therefore, we aimed to explore changes in cerebral blood flow (CBF) values in different hippocampal subfields of T2DM patients using arterial spin labeling (ASL) scan. Additionally, we examined spontaneous activity and regional functional coordination in hippocampal subfields of T2DM patients using the amplitude of low‐frequency fluctuation (ALFF) and regional homogeneity (ReHo) values of resting‐state functional magnetic resonance imaging (MRI) (rs‐fMRI). Given that coherence ReHo (Cohe‐ReHo) is more sensitive than Kendall coefficient of concordance ReHo due to its resistance to random noise caused by phase delay between measurement time (Liu et al., 2010), we selected Cohe‐ReHo as one of the rs‐fMRI metrics.

## 2 MATERIALS AND METHODS

### 2.1 Participants

We enrolled a total of 35 patients (19 males and 16 females) with T2DM according to the criteria established by the American Diabetes Association (2019). They were all treated at the First Affiliated Hospital of Guangzhou University of Chinese Medicine. Additionally, 40 healthy controls (HCs) were recruited, matched with T2DM patients in terms of sex, age, and years of education (including 23 males and 17 females). All participants were right‐handed Han Chinese and native Chinese speakers. The exclusion criteria were as follows:  (1)age <18 or >65 years old; (2) organic central nervous system disease; (3) history of mental and psychological disease and family history; (4) history of severe head trauma; (5) severe hypoglycemic history; (6) micro‐ and macrovascular complications; (7) history of alcohol dependence and poison use; (8) obvious hearing or visual impairments; (9) pregnancy, breastfeeding, and current contraceptive use (applicable to women); and (10) contraindications for MRI examination. General demographic data, including sex, age, and years of education, were self‐reported by all participants.

### 2.2 Clinical measurements

We collected systolic blood pressure (SBP), diastolic blood pressure (DBP), and body mass index (BMI) measurements from all subjects. Additionally, clinical biochemical indicators including glycosylated hemoglobin (HbA1c), fasting blood glucose (FBG), and fasting insulin (FINS) were collected from T2DM patients.

### 2.3 Cognitive evaluation

All participants underwent a series of neuropsychological assessments, including the Montreal cognitive assessment (MoCA), auditory‐verbal learning test (AVLT), clock‐drawing test (CDT), and grooved pegboard test (GPT).

### 2.4 MRI acquisition

The MRI data were acquired using a 3.0T GE SIGNA MRI scanner with an eight‐channel phased array head coil. First, we collected routine T1‐weighted, T2‐weighted, and T2‐FLAIR images as clinical MRI dataset to exclude any potential organic brain lesions. Subsequently, we conducted a quantitative evaluation of white matter hyperintensities in five regions on T2‐FLAIR images. This was done by employing the ARWMC Wahlund scoring method (Wahlund et al., 2001). Participants with a rating exceeding 2 were excluded. Two experienced radiologists, who were blinded to the group assignment, completed the score independently. In case of disagreement between the two radiologists, a joint discussion was required before the evaluation.

We then performed rs‐fMRI (Repetition Time (TR) = 2000 ms, Echo Time (TE) = 30 ms, flip angle = 90°, slice thickness = 3 mm, gap = 1 mm, Field of View (FOV) = 220 mm × 220 mm, matrixMatrix = 64 × 64, slices = 36, and measurements = 185), high‐resolution 3D T1‐weighted images (TR = 8.15 ms, TE = 3.17 ms, flip angle = 12°, slice thickness = 1 mm, FOV = 256 mm × 256 mm, matrixMatrix = 256 × 256, slices = 188, and NEX = 1), and ASL (TR = 5007 ms, TE = 10.4 ms, Number of Excitations (NEX) = 3, slice thickness = 3 mm, FOV = 240 mm × 240 mm, recon matrix = 128, arms = 8, acquisition points = 512, slices = 50) scans.

### 2.5 Image processing

We utilized GE (General Electric Company) post‐processing workstation ADW4.5 to calculate CBF image from ASL images for each subject. Then these images were registered to the standard space using the standard ASL template (MNI152_3DASL.nii) with the Matlab‐based SPM12 toolbox. The CBF value derived from ASL imaging is intimately associated with the efficiency of ASL in tissues, yet it is influenced by individual hemodynamic fluctuations. Consequently, we employed the DPABI (version 6.0) toolbox to perform Z‐transformation on the registered CBF diagram.

We used SPM8 and Restplus‐V1.2 toolboxes based on MATLAB to preprocess rs‐fMRI images. The specific steps include image format conversion, deletion of the first 10 time points, head motion correction (all subjects with head motion displacement >2 mm and head motion rotation >2° were excluded) space standardization, removal of linear drift, and regression covariables, including head motion parameters, white matter signals and cerebrospinal fluid signal noise.

Then, the ALFF and ReHo image of each subject were calculated with a frequency band of 0.01 –0.08 Hz. The Z‐transform was used to improve the normality of each metric. As hippocampal subfields were substructures with small volumes, we did not smooth the index images.

We used the SPM8‐based Anatomy Toolbox to obtain the standard spatial mask of the bilateral hippocampal subregion, including CA1, CA3, DG, hippocampus amygdala transition area (HATA), and subiculum. Then, CBF, ALFF, and ReHo values of each subject's bilateral hippocampal subfields were extracted.

### 2.6 Statistics

SPSS (IBM, SPSS, version25) was used for statistical analysis. We employed the chi‐square test to assess sex differences. Then we used the independent two‐sample *t* test or nonparametric Mann–Whitney *U* test to compare all remaining data between the groups.

We employed analysis of covariance (ANCOVA) to assess the differences in MRI metrics of bilateral hippocampal subregion between the two groups. When comparing bilateral hippocampal subregion CBF values, we used gender, age, education, SBP, and DBP as covariates. For comparison of ALFF and Cohe‐ReHo values of bilateral hippocampal subregion, we added the average head motion parameter as an additional covariate. False discovery rate was used to conduct multiple contrast correction for the results, and the significance level was set as *p* value (*Q* value) < .05 after Benjamin and Hochberg (B‐H) correction for increased reliability.

Partial correlation analysis was employed to calculate clinical laboratory indicators (HbA1c, FINS, FBG, and HoMA‐IR), cognitive tests with statistical differences (AVLT [immediate] score, CDT scores, MoCA scores, GPT [R], and GPT [L] test time), and imaging measures with statistical differences. The same covariates were used as in ANCOVA.

## 3 RESULTS

### 3.1 Demographic data and clinical laboratory indicators

The T2DM group exhibited a significantly higher BMI than the HCs group. However, there were no significant differences in sex, age, years of education, SBP, and DBP between the two groups. The detailed information is shown in Table 1.

**TABLE 1:** Demographic data and clinical biochemical indicators of all subjects.

	T2DM (*n* = 35)	HCs (*n* = 40)	Statistics (*t,x* ^2^ *,z*)	*p* value
Age (years)	51.94 ± 9.28	47.00 (44.00; 55.00)	−1.111	.266
Sex (male/female)	19/16	23/17	0.078	.780
Education (years)	12.00 (9.00; 12.00)	9.00 (6.25; 12.00)	−0.499	.618
SBP (mmHg)	119.43 ± 10.36	117.28 ± 8.08	1.010	.316
DBP (mmHg)	80.60 ± 9.21	77.50 ± 3.80	1.857	.070
BMI	24.56 ± 2.79	22.54 ± 2.51	3.304	.001*
Mean head motion parameters	0.08 ± 0.03	0.68 (0.49; 0.89)	−0.340	.734
HbA1c (%)	8.96 ± 2.01	N/A	N/A	N/A
FINS (μIU/mL)	7.94 (6.90; 8.90)	N/A	N/A	N/A
FBG (mmol/L)	7.21 (5.53; 11.30)	N/A	N/A	N/A
HOMA‐IR	2.50 (1.87; 3.74)	N/A	N/A	N/A

Abbreviations: BMI, body mass index; DBP, diastolic pressure; FBG, fasting blood glucose; FINS, fasting insulin; HbA1c, glycosylated hemoglobin; HCs, control subjects;.HOMA‐IR, homeostatic model assessment for insulin resistance; HOMA‐IR = FBG × FINS/22.5; SBP, systolic pressure; T2DM, patients with type 2 diabetes mellitus.

*p<.05, N/A, not applicable.

### 3.2 Neurocognitive test scores

The T2DM group exhibited significantly lower scores on the AVLT (immediate), CDT, and MoCA tests compared to the HCs group. Additionally, the GPT (R) and GPT (L) test times were significantly longer in the T2DM group. For cognitive test scores, please refer to Table 2.

**TABLE 2:** Neuropsychological result of two groups.

	T2DM (*n* = 35)	HCs(*n* = 40)	Statistics (*z*)	*p* value
AVLT (immediate)	19.86 ± 4.67	22.50 (18.00; 27.00)	−2.005	.045*
AVLT (5 min)	7.54 ± 2.54	7.50 (7.00; 10.00)	−0.097	.923
AVLT (20 min)	8.00 (6.00; 10.00)	8.00 (6.00; 9.00)	−0.505	.614
AVLT (recognition)	11.00 (9.00; 12.00)	11.00 (10.00; 12.00)	−0.078	.938
CDT score	3.00 (2.00; 3.00)	3.00 (3.00; 3.00)	−2.835	.005*
MoCA score	26.00 (23.00; 27.00)	27.00 (26.00; 28.00)	−2.348	.019*
GPT (R) (s)	88.40 (75.00; 100.00)	78.50 (10.00; 82.75)	−2.880	.004*
GPT (L) (s)	94.79 (79.00; 110.00)	81.30 (77.18; 90.75)	−2.433	.015*

Abbreviations: AVLT, auditory verbal learning test; CDT, the clock drawing test; HCs, control subjects; MoCA, Montreal cognitive assessment; GPT, grooved pegboard test; T2DM, patients with type 2 diabetes mellitus.

*p<.05.

### 3.3 Image characteristics of bilateral hippocampal subfields

**FIGURE 1:**
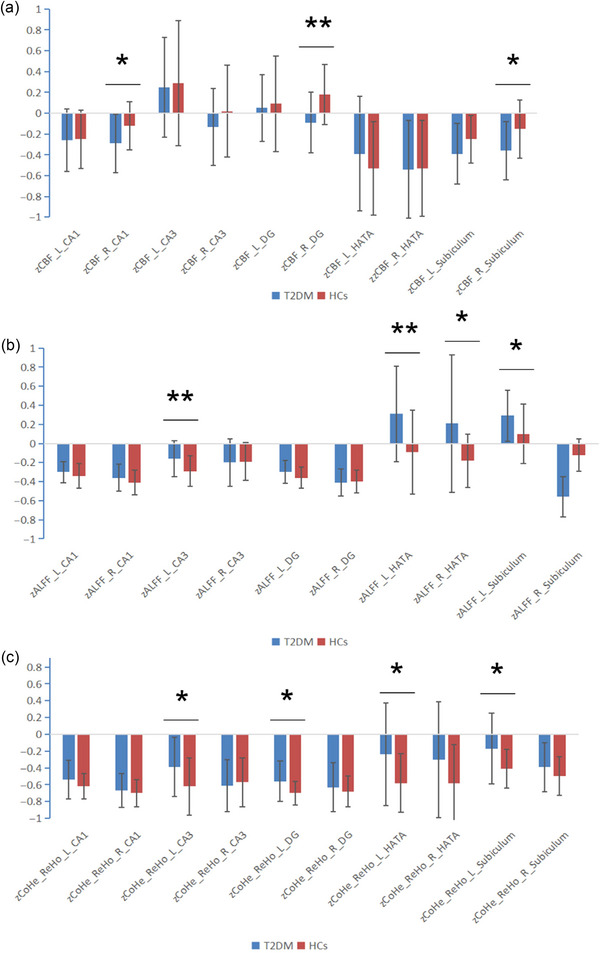
Intergroup differences in magnetic resonance imaging (MRI) metrics of bilateral hippocampal subregion. (a) Intergroup differences in cerebral blood flow (CBF). (b) Intergroup differences in amplitude of low‐frequency fluctuation (ALFF). (c) Intergroup differences in regional homogeneity (ReHo). *p <   .05, **p <   .01.

The CBF values of CA1, DG, and subiculum in the right hippocampus of T2DM group were significantly lower than those of HCs. In T2DM group, the ALFF values of left hippocampal CA3, subiculum, and bilateral HATA were higher than those of HCs. Additionally, the ReHo values of CA3, DG, HATA, and subiculum in the left hippocampus of T2DM group were higher than those of HCs (Figure 1 and Table 3).

**TABLE 3:** MRI (magnetic resonance imaging) metrics values of bilateral hippocampal subregions.

	T2DM (*n* = 35)	HCs (*n* = 40)	Statistics (*F*)	*p* value	*Q* value
**CBF values of bilateral hippocampal subregions**					
zCBF‐LH‐CA1	−0.26 ± 0.30	−0.25 ± 0.28	0.017	.897	0.997
zCBF‐RH‐CA1	−0.29 ± 0.28	−0.12 ± 0.23	7.097	.010	0.032*
zCBF‐LH‐CA3	0.25 ± 0.48	0.29 ± 0.60	0.365	.548	0.783
zCBF‐RH‐CA3	−0.13 ± 0.37	0.02 ± 0.44	3.793	.056	0.111
zCBF‐LH‐DG	0.05 ± 0.32	0.09 ± 0.46	0.045	.833	1.041
zCBF‐RH‐DG	−0.09 ± 0.29	0.18 ± 0.29	15.669	<.001	0.002*
zCBF‐LH‐HATA	−0.47 (−0.68; −0.22)	−0.64 (−0.81; −0.19)	1.423	.237	0.396
zCBF‐RH‐HATA	−0.57 (−0.79; −0.28)	−0.53 ± 0.46	0.003	.957	0.957
zCBF‐LH‐subiculum	−0.39 ± 0.29	−0.25 ± 0.23	5.533	.022	0.054
zCBF‐RH‐ Subiculum	−0.36 ± 0.28	−0.15 ± 0.28	9.413	.003	0.015*
**ALFF values of bilateral hippocampal subregions**					
zALFF‐LH‐CA1	−0.30 ± 0.11	−0.34 ± 0.13	1.493	.226	0.377
zALFF‐RH‐CA1	−0.40 (−0.45; −0.28)	−0.41 ± 0.13	1.218	.274	0.391
zALFF‐LH‐CA3	−0.16 ± 0.19	−0.29 ± 0.16	14.218	<.001	0.003*
zALFF‐RH‐CA3	−0.24 (−0.39; −0.08)	−0.19 ± 0.20	0.005	.943	0.943
zALFF‐LH‐DG	−0.30 ± 0.12	−0.36 ± 0.11	4.259	.043	0.086
zALFF‐RH‐DG	−0.41 ± 0.14	−0.40 ± 0.12	0.285	.595	0.662
zALFF‐LH‐HATA	0.31 ± 0.50	−0.19 (−0.32; 0.15)	12.560	.001	0.004*
zALFF‐RH‐HATA	−0.03 (−0.21; 0.48)	−0.18 ± 0.28	7.556	.008	0.026*
zALFF‐LH‐subiculum	0.29 ± 0.27	0.10 ± 0.31	5.814	.019	0.047*
zALFF‐RH‐subiculum	−0.56 ± 0.21	−0.12 ± 0.17	1.054	.308	0.385
**Cohe‐ReHo values of bilateral hippocampal subregions**					
zCohe‐Reho‐LH‐CA1	−0.57 (−0.68; −0.46)	−0.62 ± 0.15	4.303	.042	0.084
zCohe‐Reho‐RH‐CA1	−0.71 (−0.85; −0.51)	−0.70 ± 0.16	0.081	.776	0.776
zCohe‐Reho‐LH‐CA3	−0.39 ± 0.35	−0.67 (−0.85; −0.49)	8.963	.004	0.019*
zCohe‐Reho‐RH‐CA3	−0.66 (−0.80; −0.48)	−0.57 ± 0.29	0.330	.568	0.631
zCohe‐Reho‐LH‐DG	−0.59 (−0.71; −0.45)	−0.70 ± 0.14	9.672	.003	0.027*
zCohe‐Reho‐RH‐DG	−0.63 ± 0.29	−0.68 ± 0.18	0.635	.428	0.536
zCohe‐Reho‐LH‐HATA	−0.24 ± 0.61	−0.58 ± 0.35	8.934	.004	0.013*
zCohe‐Reho‐RH‐HATA	−0.30 ± 0.69	−0.68 (−0.91; −0.21)	3.486	.066	0.110
zCohe‐Reho‐LH‐ subiculum	−0.17 ± 0.42	−0.43 (−0.56; −0.30)	7.843	.007	0.017*
zCohe‐Reho‐RH‐ subiculum	−0.45 (−0.59; −0.23)	−0.56 (−0.65; −0.34)	1.517	.222	0.318

Abbreviations: ALFF, amplitude of low‐frequency fluctuation; CA, cornu ammonis; CBF, cerebral blood flow; Cohe‐ReHo, coherence‐based regional homogeneity; DG, dentate gyrus; HATA, hippocampus amygdala transition area; HCs, control subjects; LH, left hippocampal; RH, right hippocampal; T2DM, patients with type 2 diabetes mellitus.

*Q < 0.05.

### 3.4 Correlation analysis

**FIGURE 2:**
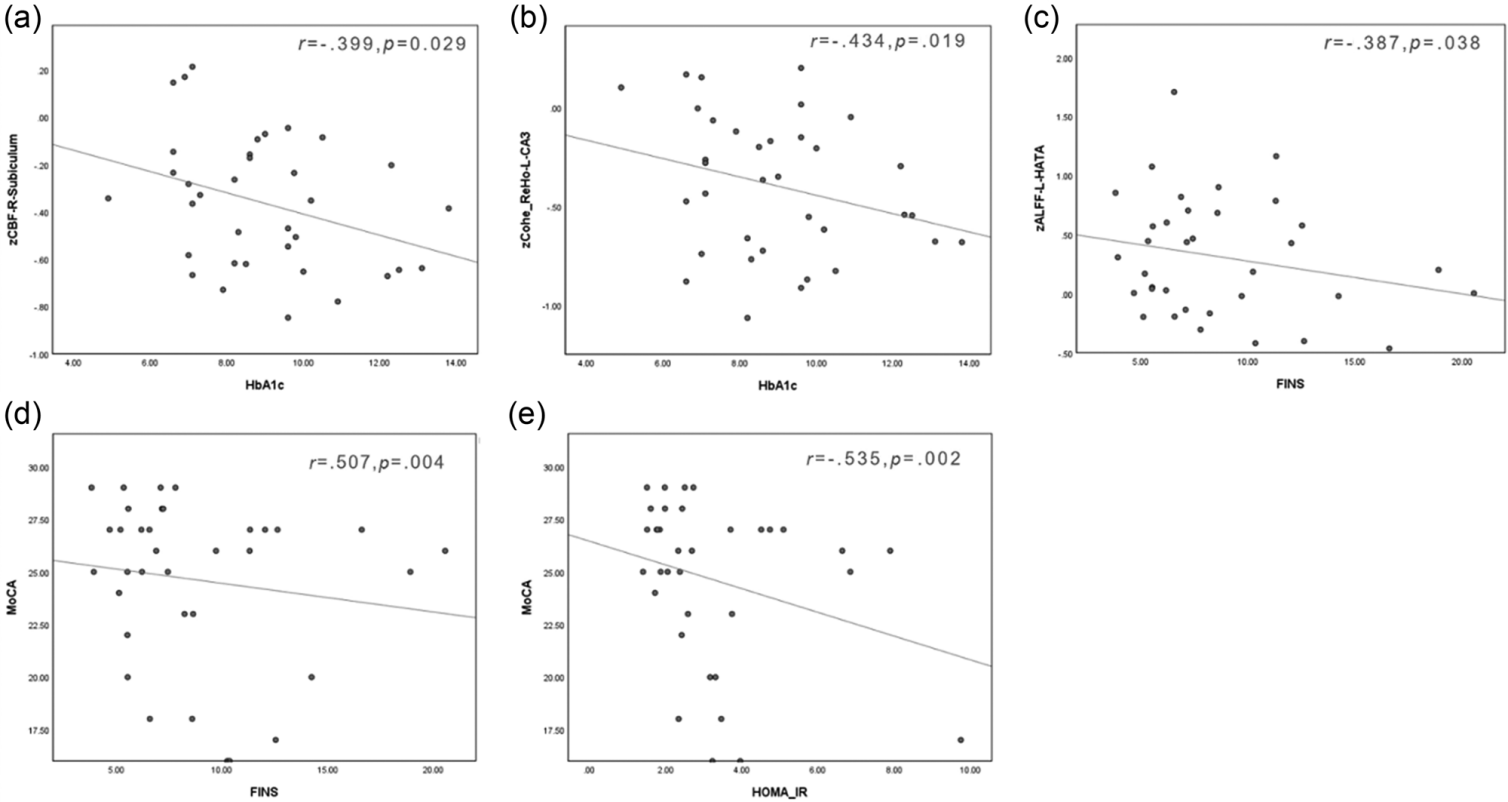
Correlation analysis. In the T2DM (patients with type 2 diabetes mellitus) group, (a) HbA1c (glycosylated hemoglobin) was negatively correlated with the cerebral blood flow (CBF) value of the right hippocampal subiculum (b) as well as the regional homogeneity (ReHo) value of the left hippocampal Cornu Ammonis area 3 (CA3). (c) Fasting insulin (FINS) was negatively correlated with the amplitude of low‐frequency fluctuation (ALFF) value of the left hippocampal HATA (hippocampus amygdala transition area). (d and e) MoCA (Montreal cognitive assessment) scores were negatively correlated with both fasting insulin (FINS) and HOMA‐IR (homeostatic model assessment for insulin resistance).

In the T2DM group, there was a negative correlation between HbA1c and the CBF value of the right hippocampal subiculum, as well as the ReHo value of the left hippocampal CA3.There was a negative correlation between FINS and the ALFF value of the left hippocampal HATA. Additionally, in the T2DM group, MoCA scores were negatively correlated with both FINS and HOMA‐IR (Figure 2).

## 4 DISCUSSION

In this study, significant reductions in the CBF values of the CA1, DG, and subiculum regions within the right hippocampus were observed in the T2DM group compared to the HCs. Furthermore, we observed a negative correlation between HbA1c and the CBF of the right hippocampus subiculum in T2DM patients. Significantly, the HbA1c concentration serves as a potent indicator of the average blood glucose level over the past 8–12 weeks, irrespective of fasting, insulin administration, hypoglycemic medications, and other influencing factors preceding the measurement. Our findings suggest that chronic hyperglycemia associated with T2DM was related to decreased perfusion in the right hippocampus subiculum. Previous research has demonstrated that chronic hyperglycemia plays a leading role in the development of vascular dysfunction and damage in individuals with T2DM, primarily involving the aldose reductase, polyol pathway (Greene et al., 1987; Kador et al., 1985), alteration of oxidation reduction potential (Tilton et al., 1992; Williamson et al., 1993), diacylglycerol‐protein kinase C pathway (Kikkawa & Nishizuka, 1986), and the accumulation of advanced glycosylation end products. Therefore, we speculate that chronic hyperglycemia induced by T2DM may cause vascular dysfunction and injury in patients, resulting in decreased perfusion in the hippocampal subregion. Although our subjects did not exhibit brain tissue damage characteristic of cerebral microvascular disease, changes in the perfusion of hippocampal microstructures are already present.

Among the hippocampal subfields prone to perfusion injury in T2DM patients, subiculum and CA1 are the main subfields responsible for information outflow from hippocampal circuits (van Strien et al., 2009). The subfields are linked to the deep layers of the entorhinal cortex and subsequently reconnected via the parahippocampal gyrus to the cerebral cortex that initially provided hippocampal input (Lavenex & Amaral, 2000). The input of CA1 pyramidal neurons (PNs) is crucial for information processing across the overall hippocampus (Masurkar, 2018). CA1 PNs possess a lengthy apical dendrite and a comparatively shorter basal dendrite, which serve as specific sites for synaptic connections with major temporal lobe pathways. The Schaffer collateral (SC) pathway is originated from CA3. In this manner, processed information from DG is carried forward to CA1 via its mossy fibers input to CA3. This completes the classical“trisynaptic pathway”from entorhinal cortex to CA1. The distal apical dendrite receives single‐synaptic input from layer III of the entorhinal cortex (EC) via the “direct pathway.” Another input to this compartment is the nucleus reuniens of thalamus, which forms a relay between prefrontal cortex and CA1. Direct inputs from CA2 mainly target the basal dendrite in *stratum oriens*, which also receives a minority of SC input. In the hippocampal memory system, CA1 neurons play a crucial role in the formation, consolidation, and retrieval of hippocampal‐dependent memories. Bartsch et al. (2011) discovered a significant impact of hippocampal CA1 damage on autobiographical episodic memory in patients, indicating that human CA1 neurons play a crucial role in retrieving remote episodic memories and also contribute significantly to autonomous consciousness. The subiculum, situated between the CA1 region and the entorhinal cortex, serves as an important conduit for information transfer in the hippocampus. It primarily receives input from the CA1 region, which is the processed information that enters the hippocampus. Additionally, the lower belt receives input from numerous cortical and subcortical regions, including a projectile from the entorhinal cortex, which is propelled by cortical activity. Consequently, the subiculum assumes a strategic position to accept and potentially integrate these two fundamentally distinct inputs. The subiculum encodes spaces in a flexible manner, contributing to the processing of allocentric information, external cues, and path integration. This supports spatial navigation extensively and plays a role in the formation of spatial memory (O'Mara & Aggleton, 2019). The DG is a critical site of neurogenesis. The neural progenitor cells, situated in the subgranular zone of the DG within the hippocampus, undergo swift amplification and differentiate into new immature neurons. These newly formed neurons migrate a short distance to the granular layer of the DG. Subsequently, these neurons mature into granule cells and are functionally integrated into the internal hippocampal circuit, receiving signal input from synapses of neurons in the entorhinal cortex via the perforation pathway, and connecting the CA3 pyramidal cells through axons to establish synaptic connections (Bruel‐Jungerman et al., 2007). Reduced perfusion in CA1, DG, and subiculum may lead to decreased hippocampal function, thus affecting the cognitive function of patients. In our study, we found that the T2DM group exhibited lower scores on AVLT (immediate), CDT, and MoCA tests than the HCs. Moreover, the time‐consuming time of GPT(R) and GPT (L) tests was longer in the T2DM group compared to the HCs. These results suggest that the memory, cognitive ability, sensitivity, and executive ability of T2DM patients were reduced, as previously demonstrated in other studies (Hoogenboom et al., 2014; Macpherson et al., 2017). Unfortunately, we did not find a simple linear correlation between reduced hippocampal subfields perfusion and cognitive decline in patients. There may be a more complex relationship between them. That requires further investigation.

In terms of rs‐fMRI, our findings indicate that T2DM patients exhibited higher ALFF values in the left hippocampal CA3, subiculum, bilateral HATA, as well as higher ReHo values in the left hippocampal CA3, DG, HATA, and subiculum, compared to the HCs. The findings suggest heightened neural activity and enhanced regional coordination within the aforementioned hippocampal subregion in individuals with type 2 diabetes. DG, CA3, and subiculum are important messaging subregions in the hippocampal trisynaptic circuit, receiving information from the entorhinal cortex and ultimately returning the information to the entorhinal cortex and a range of other cortical and subcortical areas. Functionally, DG and CA3 play a role in pattern separation and pattern completion of hippocampal memory encoding, while subiculum is involved in memory retrieval (Small et al., 2011). The HATA serves as a connection between the hippocampus and the amygdala. Previous research has established that the neural input from the amygdala plays a critical role in modulating synaptic plasticity in the hippocampus (Abe, 2001). In a recent meta‐analysis, Roesler et al. (2021) pointed out that the basolateral amygdala (BLA) affects hippocampal function through extensive amygdala‐hippocampus efflux projection (Roesler et al., 2021). Meanwhile, animal studies have shown that BLA can regulate the consolidation of memory, including spatial memory and cue discrimination memory, by affecting the hippocampus function (Packard et al., 1994; Packard & Teather, 1998). The overactivation of the hippocampus has previously been thought to be an adaptive compensatory mechanism for brain function in the early stages of cognitive impairment. This compensation mechanism is more common in Alzheimer's disease (AD) studies. Reisa's findings indicate a nonlinear trajectory of memory‐related fMRI activity during Mild Cognitive Impairment (MCI) and AD. Specifically, there is an initial phase of overactivation in presymptomatic and mild cognitive impairment subjects with memory impairment, which is followed by reduced activation as pathology and memory impairment progress (Sperling, 2007). Similarly, compensatory changes of enhanced functional connectivity in the hippocampus of patients with early stage T2DM have also been found in previous studies (Fang et al., 2019). However, previous studies have looked at the hippocampus as a unified whole, and our study further discovered how this compensatory change in the hippocampus is manifested in subregions. Some scholars also believe that the overactivity of hippocampus CA3/DG in healthy aging population may be a sign of pattern separation defect because the elderly need stronger neural activity to complete pattern separation (Yassa et al., 2011). Interestingly, in our study, the hippocampal subregion with decreased perfusion was all located on the right side, while the increased neural activity and regional coordination were mostly located on the left side. Since all our subjects were right‐handed, we speculated that the nondominant brain regions may be more prone to degeneration, while the dominant brain regions may show adaptive compensation in the process of degeneration to offset the cognitive decline to some extent. However, further research is needed to confirm this hypothesis.

In addition, in the correlation analysis, we found that HbA1c in T2DM patients was negatively correlated with the ReHo value of left hippocampal CA3, and FINS was negatively correlated with the ALFF value of left hippocampal HATA. These results suggest that although there is a compensatory increase in neural activity and coordination in the hippocampal subregion in T2DM patients, this compensatory effect still decreases with the aggravation of metabolic disorders. Therefore, the metabolic disorder of T2DM affects the neuronal activity and coordination of hippocampal subregion.

## 5 CONCLUSION

In this study, multimodal MRI was used to investigate changes in perfusion and function in the hippocampal subregion in T2DM patients. The results showed that T2DM patients had decreased perfusion in the CA1, DG, and subiculum of the right hippocampus, and the right hippocampus Subiculum was associated with chronic hyperglycemia. Additionally, we observed an increase in spontaneous neural activity within the left hippocampal CA3, subiculum, and bilateral HATA regions, as well as an enhanced local neural coordination in the left hippocampal CA3, DG, HATA, and subiculum among patients with type 2 diabetes, which may reflect an adaptive compensation for cognitive decline. However, this compensation may decline with the exacerbation of metabolic disorders.

### 5.1 Limitations

First, the course of disease was not recorded in this study. As the onset of T2DM is hidden, most patients do not know that they have T2DM before seeking medical treatment. Therefore, we acknowledge that the time of diagnosis as a measure of the disease course may not be rigorous, and we are seeking to find a better representative measure disease course. Second, the sample size in this study was small, so the conclusions are preliminary and need to be confirmed in larger studies. We will continue to expand the sample size in the future. Third, in our study, we utilize high‐resolution 3D T1‐weighted image with a voxel size of 1 mm × 1 mm × 1 mm. But a recent demonstration that the 3D T1‐weighted image resolution (1 mm × 1 mm × 1 mm) does not allow for adequate visualization of internal hippocampal structures raises concerns about the study's reliability (Wisse et al., 2021). May be this is a significant limitation in our study.

## AUTHOR CONTRIBUTIONS

**Mingrui Li**: Writing—original draft. **Yifan Li**: Writing—original draft. **Xin Tan**: Data curation. **Chunhong Qin**: Methodology. **Yuna Chen**: Software. **Yi Liang**: Writing—review and editing; Resources. **Shijun Qiu**: Resources; writing—review and editing. **Jie An**: Writing—review and editing; supervision.

## CONFLICT OF INTEREST STATEMENT

The authors declare no conflicts of interest.

### PEER REVIEW

The peer review history for this article is available at https://publons.com/publon/10.1002/brb3.3600.

## Data Availability

The data that support the findings of this study are available on request from the corresponding author. The data are not publicly available due to privacy or ethical restrictions.

## References

[brb33600-bib-0001] Abe, K. (2001). Modulation of hippocampal long‐term potentiation by the amygdala: A synaptic mechanism linking emotion and memory. Japanese Journal of Pharmacology, 86, 18–22. 10.1254/jjp.86.18 11430468

[brb33600-bib-0002] American Diabetes Association . (2019). Classification and diagnosis of diabetes: Standards of medical care in diabetes‐2019. Diabetes Care, 42, S13–S28. 10.2337/dc19-S002 30559228

[brb33600-bib-0003] Bartsch, T. , Döhring, J. , Rohr, A. , Jansen, O. , & Deuschl, G. (2011). CA1 neurons in the human hippocampus are critical for autobiographical memory, mental time travel, and autonoetic consciousness. Proceedings of the National Academy of Sciences of the United States of America, 108, 17562–17567. 10.1073/pnas.1110266108 21987814 PMC3198338

[brb33600-bib-0004] Biessels, G. , & Reagan, L. (2015). Hippocampal insulin resistance and cognitive dysfunction. Nature Reviews Neuroscience, 16, 660–671. 10.1038/nrn4019 26462756

[brb33600-bib-0005] Bruel‐Jungerman, E. , Rampon, C. , & Laroche, S. (2007). Adult hippocampal neurogenesis, synaptic plasticity and memory: Facts and hypotheses. Reviews in the Neurosciences, 18, 93–114. 10.1515/revneuro.2007.18.2.93 17593874

[brb33600-bib-0006] Cervós‐Navarro, J. , & Diemer, N. H. (1991). Selective vulnerability in brain hypoxia. Critical Reviews in Neurobiology, 6, 149–182. 1773451

[brb33600-bib-0007] Fang, F. , Lai, M. , Huang, J. , Kang, M. , Ma, M. , Li, K. , Lian, J. G. , Wang, Z. , Yin, D. Z. , & Wang, Y. F. (2019). Compensatory hippocampal connectivity in young adults with early‐stage type 2 diabetes. The Journal of Clinical Endocrinology & Metabolism, 104, 3025–3038. 10.1210/jc.2018-02319 30817818

[brb33600-bib-0008] Greene, D. , Lattimer, S. , & Sima, A. (1987). Sorbitol, phosphoinositides, and sodium‐potassium‐ATPase in the pathogenesis of diabetic complications. The New England Journal of Medicine, 316, 599–606. 10.1056/nejm198703053161007 3027558

[brb33600-bib-0009] Hoogenboom, W. S. , Marder, T. J. , Flores, V. L. , Huisman, S. , Eaton, H. P. , Schneiderman, J. S. , Bolo, N. R. , Simonson, D. C. , Jacobson, A. M. , Kubicki, M. , & Shenton, M. E. (2014). Cerebral white matter integrity and resting‐state functional connectivity in middle‐aged patients with type 2 diabetes. Diabetes, 63, 728–738. 10.2337/db13-1219 24203723 PMC3900542

[brb33600-bib-0010] Kador, P. , Robison, W. , & Kinoshita, J. (1985). The pharmacology of aldose reductase inhibitors. Annual Review of Pharmacology and Toxicology, 25, 691–714. 10.1146/annurev.pa.25.040185.003355 3923907

[brb33600-bib-0011] Kikkawa, U. , & Nishizuka, Y. (1986). The role of protein kinase C in transmembrane signalling. Annual Review of Cell Biology, 2, 149–178. 10.1146/annurev.cb.02.110186.001053 3548765

[brb33600-bib-0012] Kullmann, S. , Kleinridders, A. , Small, D. , Fritsche, A. , Häring, H. , Preissl, H. , & Heni, M. (2020). Central nervous pathways of insulin action in the control of metabolism and food intake. The lancet Diabetes & Endocrinology, 8, 524–534. 10.1016/s2213-8587(20)30113-3 32445739

[brb33600-bib-0013] Lavenex, P. , & Amaral, D. (2000). Hippocampal‐neocortical interaction: A hierarchy of associativity. Hippocampus, 10, 420–430. 10985281 10.1002/1098-1063(2000)10:4<420::AID-HIPO8>3.0.CO;2-5

[brb33600-bib-0014] Li, M. , Li, Y. , Liu, Y. , Huang, H. , Leng, X. , Chen, Y. , Feng, Y. , Ma, X. , Tan, X. , Liang, Y. , & Qiu, S. (2021). Altered hippocampal subfields volumes is associated with memory function in type 2 diabetes mellitus. Frontiers in Neurology, 12, 756500. 10.3389/fneur.2021.756500 34899576 PMC8657943

[brb33600-bib-0015] Li, Y. , Li, M. , Feng, Y. , Ma, X. , Tan, X. , Chen, Y. , Qin, C. , Huang, H. , Liang, Y. , & Qiu, S. (2021). Aberrant brain spontaneous activity and synchronization in type 2 diabetes mellitus subjects without mild cognitive impairment. Frontiers in Neuroscience, 15, 749730. 10.3389/fnins.2021.749730 34975372 PMC8716545

[brb33600-bib-0016] Li, Y. , Li, M. , Zhao, K. , Wang, Y. , Tan, X. , Qin, C. , Rao, Y. , Sun, Z. , Ge, L. , Cao, Z. , & Liang, Y. (2022). Altered dynamic functional architecture in type 2 diabetes mellitus. Frontiers in Endocrinology, 13, 1117735. 10.3389/fendo.2022.1117735 36760808 PMC9903314

[brb33600-bib-0017] Li, Y. , Liang, Y. , Tan, X. , Chen, Y. , Yang, J. , Zeng, H. , Qin, C. , Feng, Y. , Ma, X. , & Qiu, S. (2020). Altered functional hubs and connectivity in type 2 diabetes mellitus without mild cognitive impairment. Frontiers in Neurology, 11, 1016. 10.3389/fneur.2020.01016 33071928 PMC7533640

[brb33600-bib-0018] Liu, D. , Yan, C. , Ren, J. , Yao, L. , Kiviniemi, V. J. , & Zang, Y. (2010). Using coherence to measure regional homogeneity of resting‐state FMRI signal. Frontiers in Systems Neuroscience, 4, 24. 10.3389/fnsys.2010.00024 20589093 PMC2893000

[brb33600-bib-0019] Macpherson, H. , Formica, M. , Harris, E. , & Daly, R. M. (2017). Brain functional alterations in type 2 diabetes—A systematic review of fMRI studies. Frontiers in Neuroendocrinology, 47, 34–46. 10.1016/j.yfrne.2017.07.001 28687473

[brb33600-bib-0020] Masurkar, A. V. (2018). Towards a circuit‐level understanding of hippocampal CA1 dysfunction in Alzheimer's disease across anatomical axes. Journal of Alzheimer's disease & Parkinsonism, 8(1), 412. PMC6005196 29928558

[brb33600-bib-0021] McEwen, B. (1997). Possible mechanisms for atrophy of the human hippocampus. Molecular Psychiatry, 2, 255–262. 10.1038/sj.mp.4000254 9152991

[brb33600-bib-0022] O'Mara, S. M. , & Aggleton, J. P. (2019). Space and memory (far) beyond the hippocampus: Many subcortical structures also support cognitive mapping and mnemonic processing. Frontiers in Neural Circuits, 13, 52. 10.3389/fncir.2019.00052 31447653 PMC6692652

[brb33600-bib-0023] Packard, M. G. , Cahill, L. , & McGaugh, J. L. (1994). Amygdala modulation of hippocampal‐dependent and caudate nucleus‐dependent memory processes. PNAS, 91, 8477–8481. 10.1073/pnas.91.18.8477 8078906 PMC44629

[brb33600-bib-0024] Packard, M. G. , & Teather, L. A. (1998). Amygdala modulation of multiple memory systems: Hippocampus and caudate‐putamen. Neurobiology of Learning and Memory, 69, 163–203. 10.1006/nlme.1997.3815 9619995

[brb33600-bib-0025] Roesler, R. , Parent, M. B. , LaLumiere, R. T. , & McIntyre, C. K. (2021). Amygdala‐hippocampal interactions in synaptic plasticity and memory formation. Neurobiology of Learning and Memory, 184, 107490. 10.1016/j.nlm.2021.107490 34302951 PMC8435011

[brb33600-bib-0026] Schulingkamp, R. , Pagano, T. , Hung, D. , & Raffa, R. (2000). Insulin receptors and insulin action in the brain: Review and clinical implications. Neuroscience and Biobehavioral Reviews, 24, 855–872. 10.1016/s0149-7634(00)00040-3 11118610

[brb33600-bib-0027] Small, S. A. , Schobel, S. A. , Buxton, R. B. , Witter, M. P. , & Barnes, C. A. (2011). A pathophysiological framework of hippocampal dysfunction in ageing and disease. Nature Reviews Neuroscience, 12, 585–601. 10.1038/nrn3085 21897434 PMC3312472

[brb33600-bib-0028] Sperling, R. (2007). Functional MRI studies of associative encoding in normal aging, mild cognitive impairment, and Alzheimer's disease. Annals of the New York Academy of Sciences, 1097, 146–155. 10.1196/annals.1379.009 17413017

[brb33600-bib-0030] Sun, H. , Saeedi, P. , Karuranga, S. , Pinkepank, M. , Ogurtsova, K. , Duncan, B. , Stein, C. , Basit, A. , Chan, J. C. , Mbanya, J. C. , & Pavkov, M. E. (2022). IDF diabetes atlas: Global, regional and country‐level diabetes prevalence estimates for 2021 and projections for 2045. Diabetes Research and Clinical Practice, 183, 109119. 10.1016/j.diabres.2021.109119 34879977 PMC11057359

[brb33600-bib-0031] Tilton, R. G. , Baier, L. D. , Harlow, J. E. , Smith, S. R. , Ostrow, E. , & Williamson, J. R. (1992). Diabetes‐induced glomerular dysfunction: Links to a more reduced cytosolic ratio of NADH/NAD+. Kidney International, 41, 778–788. 10.1038/ki.1992.121 1513100

[brb33600-bib-0032] van Strien, N. M. , Cappaert, N. L. , & Witter, M. P. (2009). The anatomy of memory: An interactive overview of the parahippocampal‐hippocampal network. Nature Reviews Neuroscience, 10, 272–282. 10.1038/nrn2614 19300446

[brb33600-bib-0033] Wahlund, L. O. , Barkhof, F. , Fazekas, F. , Bronge, L. , Augustin, M. , Sjogren, M. , Wallin, A. , Adèr, H. , Leys, D. , Pantoni, L. , & Pasquier, F. (2001). A new rating scale for age‐related white matter changes applicable to MRI and CT. Stroke; A Journal of Cerebral Circulation, 32, 1318–1322. 10.1161/01.str.32.6.1318 11387493

[brb33600-bib-0034] Williamson, J. , Chang, K. , Frangos, M. , Hasan, K. , Ido, Y. , Kawamura, T. , Nyengaard, J. R. , Den Enden, M. V. , Kilo, C. , & Tilton, R. G. (1993). Hyperglycemic pseudohypoxia and diabetic complications. Diabetes, 42, 801–813. 10.2337/diab.42.6.801 8495803

[brb33600-bib-0035] Wisse, L. E. M. , Chételat, G. , Daugherty, A. M. , de Flores, R. , la Joie, R. , Mueller, S. G. , Stark, C. E. , Wang, L. , Yushkevich, P. A. , Berron, D. , & Raz, N. (2021). Hippocampal subfield volumetry from structural isotropic 1 mm(3) MRI scans: A note of caution. Human Brain Mapping, 42, 539–550. 10.1002/hbm.25234 33058385 PMC7775994

[brb33600-bib-0036] Yassa, M. A. , Lacy, J. W. , Stark, S. M. , Albert, M. S. , Gallagher, M. , & Stark, C. E. (2011). Pattern separation deficits associated with increased hippocampal CA3 and dentate gyrus activity in nondemented older adults. Hippocampus, 21, 968–979. 10.1002/hipo.20808 20865732 PMC3010452

